# Effectiveness of acupuncture for asthenozoospermia: A protocol for systematic review and meta-analysis

**DOI:** 10.1097/MD.0000000000025711

**Published:** 2021-04-30

**Authors:** Yuliang Zhou, Wenliang Yao, Duanjun Zhang, Yinglv Yu, Shenghui Chen, Huiyu Lu, Wanxue Jiang, Chaoren Jiang

**Affiliations:** Nanchang Reproductive Hospital, Nanchang, PR China.

**Keywords:** acupuncture, asthenozoospermia, male infertility, meta-analysis

## Abstract

**Background:**

According to the World Health Organization, the global incidence of infertility is about 15%, and more than 50% of infertility cases are caused by male infertility. Asthenozoospermia is caused by male fertility decline and male infertility. Due to work pressure, environmental pollution, sexual diseases, and other factors, the number of patients with asthenozoospermia has increased in recent years. It has been confirmed that acupuncture has a certain effect on patients with asthenozoospermia. Acupuncture and moxibustion can be an adjuvant treatment plan for the treatment of asthenozoospermia in addition to drug treatment.

**Methods:**

Randomized controlled trials of acupuncture for asthenozoospermia will be searched in the relevant database, including PubMed, Embase, Cochrane Library, China National Knowledge Infrastructure (CNKI), Wanfang Database, Chinese Biomedical Literature Database (CBM), and Chinese Scientific Journal Database (VIP database). The studies of electronic searches will be exported to EndNote V.9.1 software. We will run meta-analyses using the Review Manager (RevMan) V.5.3 software. Any disagreements will be solved in consultation with a third reviewer.

**Results:**

Our study aims to explore the efficacy of acupuncture for asthenozoospermia and to provide up-to-date evidence for clinical of asthenozoospermia.

**Conclusion:**

This study will perform a comprehensive systematic review and meta-analysis on the efficacy of acupuncture for asthenozoospermia, making up for the lack of relevant evidence of the clinical use of acupuncture.

**INPLASY registration number:**

INPLASY 202140032.

## 1 Introduction

Infertility affects about 15% of childbearing age couples worldwide, and more than 50% of infertility cases are caused by male infertility. About 1/10 of couples in China have infertility, and about 40% of them are male factors. Male infertility is related to many factors, such as genetic factors, environmental factors, reproductive tract infections, varicocele, etc.^[1,2]^ Sperm motility is vital for fertilization. The level of sperm motility is an important index for clinical measurement of male fertility. Only motile sperm can reach the fertilization site through the female reproductive tract. At the same time, survey data show that the number of sperm in male semen and various sperm activity parameters have shown a significant downward trend in the past half a century.^[3]^ With the opening of China's second-child policy and the accelerating arrival of an aging society, the Ministry of Civil Affairs has elevated the response to aging as a national strategy. In order to alleviate the pressure on the elderly, the demand for couples to have a second child has increased. Among them, there are many challenges brought about by old age, but also for men's prevention and treatment of infertility has brought great pressure.

Sperm motility is coordinated by numerous genes and various signaling pathways. At present, it is believed that asthenozoospermia is related to sperm structural abnormalities and energy deficiency.^[4,5]^ However, the causes of structural abnormality and energy deficiency can be divided into flagella,^[6]^ mitochondria,^[7]^ ion channels,^[8]^ miRNA,^[9]^ oxidative stress,^[10]^ living habits,^[11]^ environment,^[12,13]^ and other factors.^[14]^ For asthenozoospermia caused by structural abnormality, there is no other drug treatment except intracytoplasmic sperm injection for asthenozoospermia caused by energy deficiency, carnitine, arginine, and other drugs can be used.^[15]^ However, the disadvantage of this kind of drugs is that if the drugs are stopped, sperm motility will decrease.^[16]^ Due to the efficacy of a single drug cannot satisfy doctors and patients, it needs to be combined in clinical treatment. Moreover, the side effects of hormone drugs and the high cost of assisted reproductive technology have caused higher requirements for the treatment of asthenozoospermia.

As a traditional treatment method, acupuncture has been widely used in various diseases and may be a potential choice for the treatment of asthenozoospermia. In recent years, a large number of studies have shown that acupuncture is effective for various types of male infertility.^[11,17–20]^ Although there are some original documents related to the treatment of asthenozoospermia with acupuncture and moxibustion, the meta-analysis related to the treatment of asthenozoospermia with acupuncture and moxibustion is still relatively vacant. Therefore, we will use the data of all relevant randomized controlled trials (RCTs) to conduct a meta-analysis to comprehensively evaluate the efficacy of acupuncture in the treatment of asthenozoospermia.

## 2 Objectives

The aims are:

(1) to explore the efficacy of acupuncture for asthenozoospermia and
(2) to provide up-to-date evidence for clinical of asthenozoospermia.

## 3 Methods and analysis

### 3.1 Study registration

The protocol of our study is conducted in strict accordance with the Preferred reporting items for systematic reviews and meta-analysis protocols guidelines and the Cochrane Handbook.^[21,22]^ This protocol has been registered on INPLASY (registration number: INPLASY 202140032: https://inplasy.com/inplasy-2021-4-0032/).

### 3.2 Inclusion criteria

#### 3.2.1 Types of studies

All RCTs which compared acupuncture with either placebo or other drugs. RCTs conducted in adults (participants aged >18 years) without regional and language restrictions.

#### 3.2.2 Type of participants

Males diagnosed with asthenozoospermia will be considered. The diagnostic criteria for asthenozoospermia, as defined by the WHO laboratory standard, are as follows: sperm concentration <15 × 10^6^/mL and sperm motility (grade A) <32% (WHO, 2010).

#### 3.2.3 Type of interventions

The experimental group is defined as acupuncture treatment, such as manual acupuncture, warm needling moxibustion, electroacupuncture, auricular acupuncture, fire needling, elongated needle, or moxibustion.

#### 3.2.4 Types of comparators

The control group that will include non-acupuncture techniques, such as sham acupuncture, placebo, adjuvant chemotherapy, or other pharmacotherapy. The acupoint numbers, retaining time, and frequency will not be restricted in this protocol.

#### 3.2.5 Types of outcome measures

##### 3.2.5.1 Primary outcomes

The total effective rate evaluated by grade A and B sperm.

##### 3.2.5.2 Secondary outcomes

Sperm motility, sperm DNA fragmentation index (DFI), pregnancy rate, and the rate of adverse effects.

### 3.3 Exclusion criteria

Studies that are repeatedly published and necessary information cannot be obtained in various ways will be excluded.

### 3.4 Search methods for identification of studies

#### 3.4.1 Electronic searches

The electronic databases of MEDLINE, EMBASE, Cochrane Library, China National Knowledge Infrastructure Database (CNKI), Wan fang Database, China Biology Medicine Database (CBM), VIP Science Technology Periodical Database will be retrieved. We will also manually search unpublished studies and references. The search strategy that will be run in the PubMed and adjusted to fit the other database when necessary is presented in Table 1.

**Table 1:** Search strategy used in PubMed database.

Order	Search items
#1	((((((Asthenozoospermia[MeSH Terms]) OR (Astheno Teratozoospermia)) OR (Astheno Teratozoospermias)) OR (Teratozoospermia, Astheno)) OR (Teratozoospermias, Astheno)) OR (Asthenoteratozoospermia)) OR (Asthenoteratozoospermias) [All Fields]
#2	((((((((((((((((Acupuncture) OR (acupuncture therapy)) OR (warm acupuncture)) OR (fire needling)) OR (manual acupuncture)) OR (moxibustion)) OR (Acupuncture, Ear)) OR (Acupunctures, Ear)) OR (Ear Acupunctures)) OR (Auricular Acupuncture)) OR (Ear Acupuncture)) OR (Acupuncture, Auricular)) OR (Acupunctures, Auricular)) OR (Auricular Acupunctures)) OR (Electroacupuncture)) OR (electroacupuncture therapy)) OR (elongated needle) [All Fields]
#3	randomized controlled trial[Publication Type] OR randomized[Title/Abstract] OR placebo[Title/Abstract]
#4	#1 AND #2 AND #3

#### 3.4.2 Searching other resources

We will search the National Institutes of Health clinical registry Clinical Trials, International Clinical Trials Registry Platform, and ClinicalTrials.gov to find the any potentially eligible trial data.

### 3.5 Selection of studies

**Figure 1:**
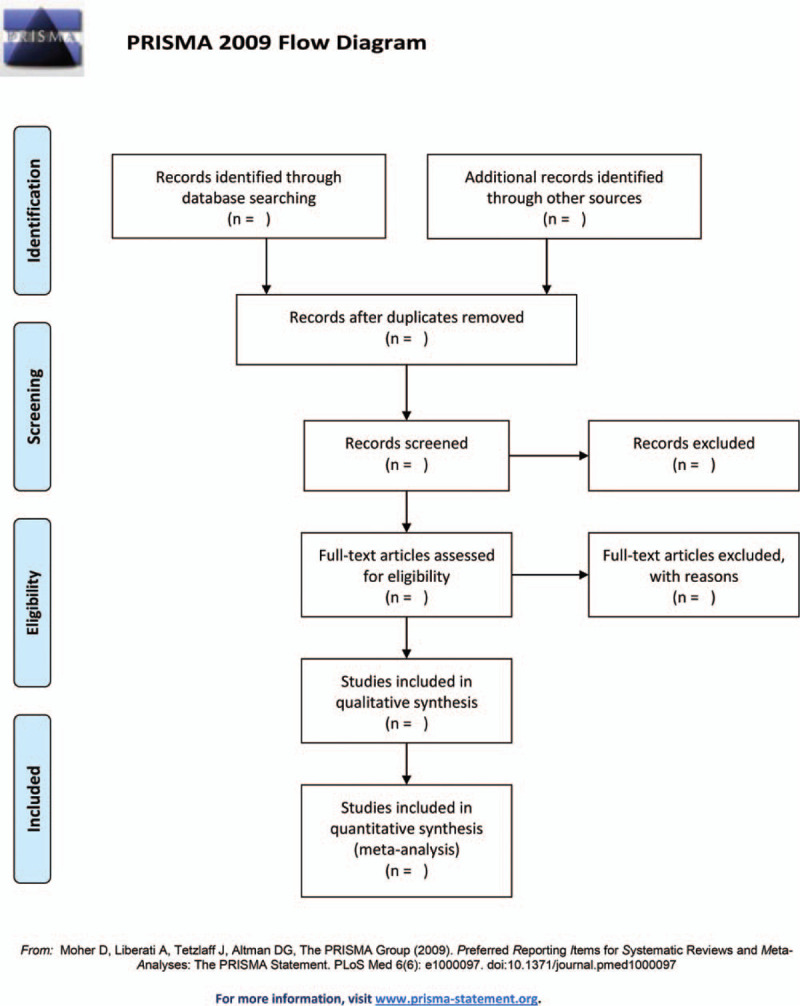
Flowchart of literature selection.

The studies of electronic searches will be exported to EndNote V.9.1 software. Two authors will independently undertake the process of selecting the search results according to the inclusion and exclusion criteria. They will review and screen the titles and abstracts retrieved by literature search to exclude irrelevant trials. The causes of both selections will be documented and full texts will be obtained and checked for further evaluation if necessary. When there is uncertainty about eligibility of the study, reviewers will arrive at a decision by via discussion and consensus with a third reviewer. The selection process will be showed in a PRISMA flow diagram (Fig. 1).

### 3.6 Data extraction and management

Data will be extracted independently from the selected articles by 2 reviewers using a Microsoft Excel spreadsheet. Information extracted from each included article will include first author, publication time, study types, characteristics of participants, type of treatments, outcome measures, and adverse events. Disagreements between reviewers in the process of data extraction were resolved by discussing with a third reviewer. Incomplete data will be provided by contacting corresponding authors.

### 3.7 Assessment of the methodological quality

The Cochrane risk assessment tool will be used by us to evaluate the methodological quality of qualified RCTs. It includes 7 items: random sequence generation, allocation concealment, blinding of participants and caregivers, blinding of outcome assessors, incomplete outcome data, selective outcome reporting, and other bias. The evaluation result of each item will be “high risk,” “low risk,” or “unclear risk.” The assessment will be completed by 2 reviewers and disagreements will be handed over to the third reviewer for the final decision.

### 3.8 Measures of treatment effect

Weighted mean difference or standardized mean difference will be adopted as statistical indicators in the analysis of continuous outcomes and the relative risk (RR) will be used to assess the treatment effect for dichotomous outcomes. 95% of the confidence intervals will be determined in pooled estimates.

### 3.9 Dealing with missing data

We will attempt to contact authors to obtain missing data. If we cannot contact the original authors, the studies will be excluded from the data synthesis.

### 3.10 Assessment of heterogeneity

Statistical heterogeneity should be evaluated by Chi-squared tests and *I*^2^ statistic. The results of the *I*^2^ statistic, which determine the using of fixed-effects model or random-effects model, cover unimportant heterogeneity (0–40%), moderate heterogeneity (30–60%), substantial heterogeneity (50–90%), and considerable heterogeneity (75–100%). A random-effect model or subgroup analysis should be used when there exists significant heterogeneity.

### 3.11 Data synthesis

Data synthesis will be completed using RevMan5.3.5 software. When *I*^2^ < 50%, we will choose the fixed-effects model; otherwise, the random-effects model will be selected. The forest plots will present the results of the meta-analyses. We will conduct descriptive analysis, when the results are not suitable for consolidation. When more than 10 studies are included, we will use the funnel plot to assess publication bias.

### 3.12 Subgroup analysis

If there is significant heterogeneity between the study results, we will perform a subgroup analysis to investigate differences in gender, age, outcome styles, etc.

### 3.13 Sensitivity analysis

We will perform sensitivity analyses to verify robustness of results. It includes the impact of methodological quality, study design, and sample size.

### 3.14 Grading the quality of evidence

Two reviewers will independently use the Grading of Recommendations Assessment, Development, and Evaluation, which evaluates the quality of evidence as “high,” “moderate,” “low,” or “very low,” to assess the quality of evidence.^[23]^

### 3.15 Ethics and dissemination

The study will be published in a peer-reviewed journal or relevant conference. No ethical approval is required. The results of the study will provide potential guidance in advancing the therapeutic strategy of patients with asthenozoospermia.

## 4 Discussion

Acupuncture for asthenozoospermia is a microtrauma surgery with less pain. So, it is crucial to make sure whether acupuncture is a good option for patients. The previous studies have indicated that acupuncture can effectively improve sperm motility; however, its efficacy has not been assessed scientifically and systematically. To address this limitation, this study will inspect the efficacy and safety of the acupuncture in patients with asthenozoospermia. This review also has some limitations. The different acupoints of acupuncture and the different severity of asthenozoospermia may induce the heterogeneity.

## Author contributions

**Conceptualization:** Yuliang Zhou, Shenghui Chen.

**Data curation:** Yuliang Zhou, Duanjun Zhang, Yinglv Yu.

**Formal analysis:** Wenliang Yao, Huiyu Lu, Wanxue Jiang.

**Methodology:** Wenliang Yao, Yinglv Yu, Chaoren Jiang.

**Software:** Duanjun Zhang, Huiyu Lu, Wanxue Jiang.

**Supervision:** Duanjun Zhang, Shenghui Chen.

**Writing – original draft:** Yuliang Zhou, Duanjun Zhang, Yinglv Yu.

**Writing – review & editing:** Shenghui Chen, Huiyu Lu, Wanxue Jiang.
